# First Drinking Experiences during Adolescence in South Korea: A Qualitative Study Focusing on the Internal and External Factors

**DOI:** 10.3390/ijerph18158200

**Published:** 2021-08-03

**Authors:** Seong-Jun Maeng, Dong-Jun Lee, Jun-Hyeok Kang

**Affiliations:** 1 Department of Social Welfare, Sungkyunkwan University, Seoul 03063, Korea; bossmsj@skku.edu (S.-J.M.); leedj1992@skku.edu (D.-J.L.); 2 Department of Addiction Rehabilitation and Social Welfare, Eulji University, Seongnam 13135, Korea

**Keywords:** alcohol, first drinking experience, South Koreans adolescents, case study

## Abstract

This study explored the internal and external factors affecting the first drinking experience during Korean adolescence. To achieve this, we collected 34 cases revealing specific drinking experiences during adolescence in Alcoholic Anonymous (A.A.), Korea. The collected data were analyzed using a qualitative case study method, and the analysis focused on the internal and external factors influencing drinking in adolescence. As per the results, internal factors that influenced drinking in adolescence were “curiosity” and “elevated mood and stress relief”, and external factors were “family”, “friends”, “older friends at school”, “neighbors”, “Korean tradition of alcohol making”, “workplaces that encourage alcohol consumption”, and “a generous drinking culture.” Based on these findings, we suggested several practical alternatives, such as a stringent alcohol punishment system, government-led campaigns to overcome the generous alcohol culture, monitoring the drinking status of working and intern youths, and using local crime prevention guards to curb youth drinking. In future research, it is necessary to quantitatively verify the results of this study to develop theories related to adolescent drinking behavior.

## 1. Introduction

Excessive drinking causes several problems. Individuals who drink excessively may experience physical and mental problems and accidents such as drunk driving [1]. Additionally, health deterioration caused by drinking also incurs social costs such as medical and nursing care expenses [2]. Moreover, Korea’s generous drinking culture and alcohol advertising further support drinking [3]. In Korea, people can freely buy and drink alcohol at any time. In addition, Korea uses the AUDIT scale, which is a measure related to alcohol use, in a more relaxed manner compared to other countries.

The social service sector deals with alcohol abuse problems in all age groups, but we need to pay more attention to adolescents’ drinking problems. This is because adolescent drinking behavior can lead to other delinquent behaviors [4]. In many countries including Korea, it is illegal for adolescents to buy or drink alcohol. According to the Youth Protection Act in Korea, people over the age of 19 can purchase alcohol. Some of them commit crimes such as forging ID cards or theft in order to drink alcohol. For example, in Korea, food delivery services have been activated due to COVID-19, making it a social issue because adolescents purchase alcohol using their parents’ information [5]. Moreover, because adolescence is a period of severe emotional fluctuations, drinking alcohol is highly likely to lead to destructive behaviors that threaten their and others’ lives [6,7,8].

Adolescence is also a period of rapid physical growth; the risk of damage to organs such as the brain, liver, and kidneys, and sexual dysfunction due to alcohol consumption is higher than that in adults [1]. Constant alcohol consumption causes alcoholism. In adolescence, because self-control is not yet completely developed, adolescents tend to become addicted to alcohol more quickly than adults [9]. Particularly, consuming alcohol in adolescence can lead to alcohol use disorders in adulthood because the lower the drinking age, the greater the likelihood of developing tolerance to alcohol [10,11]. For these reasons, drinking negatively affects adolescents and is recognized as a major cause of injuries and deaths worldwide [12]. In Korea, the age of first-time drinkers is decreasing. According to the Korean Ministry of Gender Equality and Family, the age of first drinking experience among adolescents in 2018 was 13.0 years for males and 13.7 years for females, but in 2019, it was lowered to 12.9 years for males and 13.5 years for females. Therefore, drinking problems among teenagers are considered to be more serious than those of other age groups [13]. Further interventions are needed to prevent and solve adolescent drinking problems.

To better understand and intervene in the drinking experience of adolescents, the individual and the surrounding environment must be considered together. Ecological systems theory emphasizes the understanding of interactions between humans and the environment [14] and regards humans as active and sociocultural beings. According to this theory, humans respond actively to the demands of the environment and adapt, sometimes changing the environment to suit them [15]. In other words, ecological systems theory argues that it is impossible to understand humans and the environment separately and that humans and the environment must be considered together. Ecological systems theory does not only emphasize the environment surrounding humans. This theory suggests that personal factors such as biological, psychological, and spiritual factors are also important [14]. Therefore, we classified factors affecting adolescents’ drinking into internal and external and looked at previous studies based on this distinction.

Demographic factors such as gender and age can be considered internal factors affecting adolescents’ drinking. In general, the drinking rate in men is higher than that in women, and it increases with age [2,8,16,17]. Psychological factors such as thrill-seeking behavior, impulse control problems, curiosity about alcohol, lack of knowledge about drinking, and expectations about drinking influence adolescent drinking [18,19,20,21,22,23]. Mental-health-related factors include emotional anxiety, stress, depression, and hopelessness [24,25]. In general, adolescents with higher levels of stress or depression drink more than those without with lower levels [2]. According to a related study, adolescents who engage in health risk behaviors such as drugs and cigarettes are more likely to drink than those who do not [6].

Environmental factors include family, peer group, school, and culture. Reportedly, relations with family and family history affect adolescents’ drinking habits. Dysfunctional interactions within the family, such as conflicts, neglect, and inconsistent parenting attitudes have been reported to be closely related to drinking in adolescents [26,27]. Parents’ drinking is also related to adolescent drinking. If parents continue to drink, their interest in their children reduces, and parenting attitudes may become negative, leading to an increase in their children’s drinking. Children are more likely to drink alcohol by learning their parents’ drinking behavior [28,29].

Because adolescents are influenced by peer groups, drinking and peer pressure are important variables predicting adolescents’ drinking [21,22]. Particularly, the delinquency of adolescents at a younger age is mainly caused by parental influence, but the deviant behaviors that adolescents exhibit as they grow are related to their peer groups [30,31]. Adolescents drink alcohol as a rite of passage to fit in with their peer group, and exchange and learn information, such as purchasing alcohol and drinking culture. Through this process, adolescents move away from healthy peer groups, become more intimate with their drinking peers, and drink more often. As a result, drinking alcohol can lead to other types of misconduct, such as a decrease in academic achievement and runaway. Additionally, academic failure due to a low level of academic achievement affects adolescents’ drinking [32,33]. Particularly, Korean society with its strong educational enthusiasm tends to evaluate adolescents based on their academic performance. Considering these cultural factors, it can be said that the drinking problem of adolescents was created by society.

Sociocultural factors have also been reported as predictors of drinking in adolescents. It was found that the price of alcohol and the drinking rate of adolescents were inversely proportional [34], and the higher the ease of purchase of alcoholic beverages, the higher the likelihood of drinking alcoholic beverages [2,35]. Additionally, the environment in which adolescents live affects their drinking. The drinking rate of adolescents living in communities with high adult drinking rates is also high [34,36]. Alternatively, excessive media exposure and alcohol advertisements may increase the teenage drinking rate [3].

Previous studies presented above suggest that various factors influence adolescents’ drinking. However, they have some limitations. First, prior studies did not address the causes of alcohol initiation in adolescents. Additionally, existing studies mainly analyzed quantitative data to identify factors that influence adolescents’ drinking, so they did not reveal specific drinking contents and experiences. To overcome these limitations, we focused on why the first drinking experience occurred in adolescence. Therefore, we collected cases related to the first drinking experience of adolescents and tried to determine the internal and external factors that act as the first drinking trigger. Through this process, it is possible to contribute to the establishment of prevention strategy by specifically revealing the drinking pathways of adolescence.

## 2. Materials and Methods

### 2.1. Sampling and Data Collection

We used a purposeful sampling method for data collection because it was necessary to select cases that fit the purpose of the study and to explore adolescents’ first drinking experience. We collected alcoholic recovery notes published from January 2015 to March 2021 in Alcoholic Anonymous (A.A.), Korea, to identify cases of adolescent drinking experiences. A total of 236 cases of actual experiences were secured, and among them, 34 cases were analyzed as they contained first alcohol experiences during adolescence in detail. In our data, alcohol consumers in adolescence drank various types of alcohol between the ages of 5 and 18 and were influenced by internal and external factors such as curiosity and recommendations from friends (Table 1).

### 2.2. Data Analysis

We used a qualitative case study method to analyze the collected data [37]. Qualitative case study method enables detailed explanations of a specific phenomenon, unlike a quantitative study that focus on causality verifying. Especially, this method is suitable for extracting themes of discussion based on the commonalities of the first drinking experiences in adolescence. Additionally, it enables the comparison of results with existing knowledge [37]. By checking whether the research results are consistent with existing knowledge, we can expand the scope of our knowledge or present grounds for revealing that existing ideas are false. Through this process, a rich interpretation of the first drinking experience in adolescence is possible. Specific research procedures are as follows; first, reading the recorded contents to find meaningful units. This step is similar to open coding in grounded theory. Second, separating necessary and unnecessary data. This step is the step of extracting important data related to the research topic through continuous comparative analysis. Third, finding relationships between codes or categories to form a central theme. In this step, the central topic for discussion is confirmed by checking the association between codes. Lastly, linking research results with existing knowledge. This step expands the scope of existing knowledge by asking the questions presented in the table below (Table 2).

### 2.3. Ethics and Rigor

To conduct qualitative research, the rigor of research and ethical issues should be considered together. Because this study analyzed secondary data called casebooks, ethical issues that might have otherwise risen during interviews or observations did not arise. Therefore, we focused on securing the rigor of the study. To do so, we used strategies such as triangulation, peer support groups, and audit trails [37]. First, we used theory and analyst triangulation strategies. For theory triangulation, the factors influencing adolescents’ drinking, as suggested by previous studies, were reviewed and compared with our study results. For analyst triangulation, each researcher analyzed the data and compared them, and recognized only the agreed-upon analysis results as a common theme. Second, we organized an addiction rehabilitation and one youth counseling expert into a peer group and sought their advice on the research. Third, we conducted an audit trail to increase the reproducibility of the study. To this end, we retained data such as memos and category lists written during the study so that subsequent researchers can trace the process and derive similar results.

## 3. Results

As a result of the analysis, two internal factors were found, including “Curiosity” and “Elevated mood and stress relief”. External factors were found, including “Family (Parents, Grandparents, Cousins)”, Friends”, “Older friends at school”, Neighbors (adult)”, “Korean tradition of alcohol making”, “Workplaces that recommended alcohol” and “Generous drinking culture”.

### 3.1. Curiosity

Curiosity acted as an internal factor in that those who drank alcohol in adolescence drank out of curiosity. As suggested by previous studies, curiosity serves as a motive for drinking alcohol during adolescence [20,22]. Some alcohol users (cases 1, 2, and 3) first drank alcohol out of curiosity when they were in high school, and this became a factor for repeated drinking.

“I remember drinking for the first time when I was in the freshman of high school. I secretly drank out of curiosity with my friends, and when I became an adult, I drank every day with my acquaintances.” (case 1)

“The first time I drank alcohol was when I was 17 years old. At first, I started drinking beer out of curiosity, and after a few months, I drank *soju*.” (case 2)

“The first time I drank alcohol, I was in the freshman of high school, and I drank out of curiosity. After drinking, I felt like I owned the whole world, and I felt a sense of freedom from not having to hide myself anymore.” (case 3)

### 3.2. Elevated Mood and Stress Relief

Those who experienced drinking alcohol in adolescence did not drink simply out of curiosity. Alcohol served as a tool to elevate their mood and relieve stress. Previous studies have also noted that emotional factors such as emptiness, emotional instability, thrill-seeking behavior, and impulse control problems are related to drinking in adolescence [2,23,25]. In this study, the details of the emotional factors were identified. Adolescent alcohol users felt better and bolder after drinking (cases 3, 4, 5), and some felt that it relieved their psychological stress such as lethargy, anxiety, and depression (cases 6, 7, 8).

“After drinking, I felt like I owned the whole world, and I felt a sense of freedom from not having to hide myself anymore. I was afraid of people because I had an introverted personality. However, I was not afraid anymore.” (case 3)

“When I drank, I kept smiling and felt very good, and I felt my usual introvert personality became bold.” (case 4)

“The first time I drank alcohol was when I was 16 years old. A relative ran a liquor dealership in the neighborhood, when I had the time, I went to work there. There, I learned that drinking made me feel good, and I started to drink more and more.” (case 5)

“I had a conflict with my friend in middle school, but I was upset that I could not resolve it and could not get angry properly. I suffered from lethargy, and my academic performance decreased. So, I drank for the first time when I was in the sophomore of middle school.” (case 6)

“When I drank alcohol, my nervousness, anxiety, and frustration seemed to disappear. Alcohol cured me faster than medicine.” (case 7)

“When I was 16 years old, my close friend died, and my parents broke up. I was very shocked. So, that’s when I started wandering. I drank with my friends for the first time, and alcohol caused my stress, irritation, depression, and anxiety to disappear.” (case 8)

### 3.3. Family (Parents, Grandparents, Cousins)

The old Korean maxims have a saying, “Drinking should be learned from adults,” meaning adults in the household teach drinking etiquette, which shows the family’s influence on adolescent alcohol drinking. Of course, existing studies also point at “family” as a factor in drinking alcohol in adolescence. However, previous studies have shown that dysfunctional family relationships (family conflict, parental neglect, dysfunctional communication, and inconsistent parenting attitude) lead to drinking [22,26,27,29]. On the other hand, in this study, it was confirmed that the culture of recommending alcohol to families in the absence of conflict causes alcohol consumption in adolescents. In this study, alcohol users began drinking alcohol in adolescence at the recommendation of their cousins, parents, and grandparents (cases 9, 10, 11, 12). This is related to a Korean government report that confirmed that 45.3% of the 3626 teenagers surveyed had been recommended to drink at restaurants by their families [38].

“I started drinking in earnest when I was a sophomore in high school. A relative taught me how to smoke cigarettes and drink alcohol.” (case 9)

“I had a good relationship with my dad, and when I was 5, my dad gave me the leftover alcohol, and I drank it pleasantly.” (case 10)

“My family had many Korean ancestral rites, so I was lucky as I drank alcohol during Korean ancestral rites every time. Family who saw me drinking thought I was cute, so they recommended me to drink more.” (case 11)

“I first started drinking when I was 15 years old, and my grandfather suggested that I drink.” (case 12)

### 3.4. Friends

In Korea, a friend refers to a person of the same age. In Korean culture, where age is important in relationships, people tend to communicate more comfortably with other people of the same age. In previous studies [18,21,22], “friends” were also seen as a drinking factor in adolescence. Previous studies have shown that adolescents drink alcohol owing to peer pressure, to fit in with their peers (passing rites), or to imitate feelings toward friends. These factors were also observed in the present study. However, in this study, alcohol users naturally drank alcohol while hanging out with friends rather than drinking owing to peer pressure.

“When I was in the sophomore of high school, I drank with my friends in the mountains in the neighborhood, calling it a rite of passage.” (case 11)

“When I was 18 years old, I drank a large amount of alcohol with my friends for the first time.” (case 13)

“When I was in the sophomore high school, I drank *makgeolli* with my friends during the winter vacation and vomited a lot. I rarely talked to my friends when I did not drink.” (case 14)

“The first time I drank alcohol was in the fall when I was 15 years old. I had three bottles of *soju* with my friends at a park in the village and drank yogurt as a snack.” (case 15)

“I drank for the first time when I was in the sophomore of middle school. I hated studying and naturally hung out with juvenile delinquents.” (case 16)

“I drank for the first time when I was in the junior of middle school. I liked to play with my friends and hated studying.” (case 17)

“When I was in the junior of high school, I drank with my friends for the first time. I felt that the draft beer I drank with my friends for the first time tasted worse than coke, but I drank it at once seeing my friends drink.” (case 18)

### 3.5. Older Friends at School

The recommendation and coercion of older friends at school also served as a trigger for drinking alcohol during adolescence. As mentioned earlier, in Korean society, where age is important for the formation of relationships, high and low authority relationships are formed around the same age [39]. Particularly, in educational groups such as schools, the higher the grade, the higher the authority. Due to this power structure, juniors take the advice or coercion of their older friends at school. Alcohol users in this study also drank at the recommendation of their older friends at school (cases 19, 20, 21, 22) while studying in middle school, high school, university (case 23), and because they could not overcome the coercion of their older friends (case 23).

“I did not think that the drinking habits I learned from older friends in middle school would be the trigger for alcoholism until I was 44 years old.” (case 19)

“The first time I drank alcohol was when I was 15 years old. I went to my friend’s house and drank the beer that my friend’s sister bought for the first time. Dancing drunk all night made me feel better.” (case 20)

“The first time I drank alcohol was when I was in the sophomore of middle school. My friends and I drank because older friends at school told us to come to drink.” (case 21)

“The first time I drank alcohol was when I was in college, and I drank *makgeolli* recommended by older friends at school in celebration of the freshmen.” (case 22)

“When I was in the second year of middle school, older friends at school ordered me and my friends to buy alcohol. Older friends started drinking, then assaulted me and my friends who did not drink with the club, forcing us to drink.” (case 23)

### 3.6. Neighbors (Adult)

Along with family, friends, and older friends at school, neighbors also influence alcohol drinking in adolescence. Adolescent alcohol users drank alcohol as recommended by local adults that they were close to (cases 4, 6, 24). These examples specifically illustrate the government’s findings that 24.6% of the 3595 teenagers surveyed were recommended to drink by adults outside their families at restaurants [38]. Although it is illegal to sell alcohol to teenagers in Korea, it is not illegal to recommend alcohol; hence, alcohol can be recommended without guilt. This shows that adults who naturally recommend alcohol influence adolescent drinking.

“I had my first drink when I was in elementary school. The man who owned the laundromat offered me a shot of *makgeolli*. Alcohol overwhelmed my small body and immature mind.” (case 4)

“When I was a senior in high school, a local man gathered the kids and made them drink. I drank until I was blacked out.” (case 6)

“I first drank alcohol during the autumn harvest of rice farming in 12 years old. The elders who had been eating *makgeolli* were excited and poured into my bowl. I was hungry and thirsty, and the *makgeolli* was just the drink and food for me.” (case 24)

### 3.7. Korean Tradition of Alcohol Making

In the past, many families in Korea made their own alcohol [40]. Although most of them disappeared due to the strict control of homemade alcohol by the State Tax Act of 1907, the Korean tradition of alcohol making (homemade) still exists. Some adolescents (cases 25 and 26) secretly drank alcohol made by their mother or grandmother at home. These examples show that making or storing alcohol at home naturally provides adolescents with an opportunity to drink.

“When I was a middle school student, I stole alcohol, which my mom soaked from home and drank. My face burned red, and an indescribable ecstasy came to me, and I drank whenever I had a chance.” (case 25)

“I saw my grandmother drinking after working on a hot summer day when I was in elementary school. She drank homemade *makgeolli* with sugar. I drank it secretly, and every time I drank it, I would wander around and fell asleep.” (case 26)

### 3.8. Workplaces That Encourage Alcohol Consumption

Most students who drop out of school or attend vocational high schools find employment in early jobs or internships. During this process, people encountered alcohol (cases 27, 28, 29, 30, 31, 32). As they watched adults working together drink alcohol, they naturally imitated it, and also drank at the recommendation of adults. This suggests that even adolescents can be easily exposed to alcohol if they live together in a space centered on adults. Additionally, like in case 31 that “Even if you are young, you are adult when you are work!”, the possibility of drinking at work increases if you are recognized as an adult.

“When I was a senior in high school, I went on vocational training. It started then. I was exposed to alcohol easily because I was a student and worker. Alcohol was my friend in difficult situations where I had to work overtime.” (case 27)

“I decided to give up on graduating from high school and learned vocational skills. As I was working, I always saw people drinking the *haejangsul* (alcohol for lessening the effect of a hangover) in a workplace restaurant in the morning. I drank too much a day ago, but after drinking the *haejangsul*, I felt like I would live.” (case 28)

“At a young age (from a senior high school), I ran away from home and boarded a tuna fishing ship. Then, for the first time on a ship, I learned to drink liqueur.” (case 29)

“When I was 16, I dropped out of school and got a job at a factory. While hanging out with seniors, I started drinking for the first time at the age of 19.” (case 30)

“At the age of 17, I had to drop out of high school after several months due to difficult family conditions. I got a job as a deliveryman at a Chinese restaurant and started to follow the grown-ups by having a drink or two. Adults said, ‘Even if you are young, you are an adult in social life!’ Then, they gave me a drink.” (case 31)

“I drank for the first time when I dropped out of high school as a sophomore and started working in a factory. At first, after the night shift, I could not sleep, so I drank a glass or two at the recommendation of my senior colleagues.” (case 32)

### 3.9. Generous Drinking Culture

As mentioned in several previous studies [41,42,43], the culture of generously drinking alcohol is also closely related to drinking. A generous drinking culture acts as a drinking factor in adolescents. Similar results were observed in this study. Adolescents with alcohol use noted that this culture brought them closer to alcohol. They learned about alcohol while running errands at home (case 10), and there was no reluctance to drink because it seemed natural in adolescence (case 33). Particularly, due to the generous culture, “Mistakes were made by alcohol, not by human.” Caused by alcohol, we were able to drink without much burden.

“The wrong drinking culture and tolerance for alcohol made me alcoholic. From junior high school days, when I was running errands, it was fun to hold the *makgeolli* kettle in my mouth.” (case 10)

“A bottle of soju, which I started drinking at the age of 16, led me to addiction. I thought big and small mistakes were caused by alcohol and not by me.” (case 33)

“Drinking *makgeolli* or beer during adolescence is a natural idea. Many people drank that way.” (case 34)

## 4. Discussion

In summary, curiosity, elevated mood, and stress relief were internal factors that enabled adolescent drinking, and family (parents, grandparents, cousins), friends, older friends at school, adult neighbors, Korean tradition of alcohol making (homemade), workplaces that recommended alcohol, and generous drinking culture were the external factors. This is illustrated in Table 3.

Compared to existing studies, the internal factors identified in this study were similar to those identified in the existing studies. However, school seniors, neighbors (adults), drinking culture, and workplaces encouraging alcohol were not pointed out in previous studies but served as a trigger for drinking in adolescence. The commonality of these factors is that older drinkers recommended or forced alcohol. From an ecological systems theory perspective, an individual adapts by interacting with surrounding environmental systems, such as family and neighbors [14]. To adapt, adolescents sometimes accept their suggestions and imitate their actions.

### 4.1. Limitations

Although this study discovered internal and external factors that influenced Koreans’ first drinking experience in adolescence, there are several limitations. First, because we extracted data from the alcoholic recovery notes, we could not examine phenomena other than the contents in the notes. Additionally, the contents of the collected data were written around past experiences. The research participants were unable to look at a series of changing life processes because the data were not collected during adolescence. Therefore, in future research, it is necessary to specifically explore the drinking experience of adolescence through 1:1 in depth interviews. Second, this study does not have quantitative figures. Thus, it is not shown the tendency of drinking experience among adolescents. Therefore, in future research, it is necessary to confirm the tendency of adolescent drinking through quantitative research. A follow-up study can be conducted based on these limitations.

### 4.2. Practical Implications

First, strengthening the regulation of adolescent drinking is necessary. This study found that adolescent drinkers were influenced by adults and older friends at school, who recommended alcohol. Currently, in Korea, only restaurant operators who sell alcohol to teenagers are punished. To improve the system, it is also necessary to consider punishment for adults who recommend and youth who drink alcohol. In case of difficulty in amending the law, it is necessary to revise school regulations to manage the consumption and recommendation of alcohol.

Second, a government-led campaign is needed to overcome the generous alcohol culture. To solve this problem, a government-led campaign to stop drinking alcohol is needed, such as the smoking cessation campaign. Previously, Korea experienced a reduction in the smoking rate of citizens through active government-led anti-smoking campaigns. To change the tolerant drinking culture that negatively affects youth, various activities such as TV advertisements, mandatory education on the dangers of drinking, and counseling support through community health centers should be carried out by applying the smoking cessation campaign model. Particularly, the culture of making and drinking alcohol at home was also related to drinking opportunities during adolescence. Therefore, it is necessary to take this into account when campaigning. The culture of making alcohol at home should be protected because it is a traditional Korean culture. Even so, because homemade alcohol is also alcohol, the government needs to guide parents so that they can be aware of this and pay attention to its storage.

Third, the status of early employment and youth training should be checked. Results showed that young people in early employment and industrial training were more exposed to alcohol by hanging out with adults at work. Considering these factors, a survey should be conducted on the drinking conditions of working youth. Based on this, it is necessary to develop a strategy to prevent alcohol consumption by youth who work in industries.

Fourth, it is necessary to use a local crime watch. As a result of the study, those who experienced drinking alcohol in adolescence drank at local hills and parks. In Korea, there is a network of local crime watches. It is necessary to make the most of this and manage adolescents who drink outdoors at night.

Finally, an integrated approach to the adolescents’ drinking problem is needed. As a result of the study, various factors, internally and externally, were influencing adolescent drinking behavior. Therefore, mental health experts need to take a comprehensive look at the drinking problem of adolescents by considering various internal and external factors at the same time.

## 5. Conclusions

The purpose of this study was to explore the factors that affect adolescent alcohol drinking. The study found that the internal factors that influenced drinking in adolescence were curiosity, elevated mood, and stress relief, and external factors included family, friends, older friends at school, neighbors, Korean tradition of alcohol making, a workplace that recommended alcohol, and generous drinking culture. Among these, the newly identified factors were older friends at school, the neighbors, Korean tradition of alcohol making, and the workplace that recommended alcohol.

We understood the findings from an ecological systems theory perspective that emphasizes the interaction between individuals and the environment. Therefore, to understand the drinking of adolescents, it is necessary to consider not only the individual’s internal motivation but also the ecological environment that affects it.

## Figures and Tables

**Table 1:** Case description.

Case Number	Gender	First drinking Experience Time	Type of Alcohol	Route
1	unknown	High school student period	unknown	curiosity
2	male	High school student period	Beer, *soju*	curiosity
3	male	High school student period	unknown	curiosity
4	male	Elementary student period	Rum, *makgeolli*	Uplifted mood, Neighborhood (adult)
5	male	Middle school student period	*makgeolli*	Uplifted mood
6	female	Middle school student period	*soju*	Relieve stress, Neighborhood (adult)
7	female	High school student period	Beer	Relieve stress
8	female	Middle school student period	unknown	Relieve stress
9	male	High school student period	unknown	Cousin
10	male	5 years old period	*makgeolli*	Father, Generous drinking culture
11	male	Elementary student period	*Korean Traditional alcohol*	Family
12	male	Middle school student period	unknown	Grandparents
13	male	High school student period	*soju*	Friend
14	male	High school student period	*makgeolli*	Friend
15	male	Middle school student period	*soju*	Friend
16	male	Middle school student period	*soju*	Friend
17	male	Middle school student period	unknown	Friend
18	male	High school student period	Beer	Friend
19	unknown	Middle school student period	*soju*	Senior at school
20	female	Middle school student period	Beer	Senior at school
21	female	Middle school student period	Beer	Senior at school
22	female	College student period	*makgeolli*	Senior at school
23	male	Middle school student period	*soju*	Senior at school
24	male	Elementary student period	*makgeolli*	Neighborhood (adult)
25	male	Middle school student period	Soaked Liquor	Culture of soaked Liquor
26	male	Elementary student period	Soaked Liquor	Culture of soaked Liquor
27	male	High school student period	*soju*	Workplace
28	male	High school student period	*soju*	Workplace
29	male	Middle school student period	whiskey	Workplace
30	male	High school student period	*soju*	Workplace
31	male	High school student period	*soju*	Workplace
32	male	Middle school student period	unknown	Workplace
33	male	High school student period	*soju*	Generous drinking culture
34	male	High school student period	*makgeolli*, Beer	Generous drinking culture

In Korea, the age of elementary school students is 7–12 years, middle school students 13–15 years, and high school students 16–18 years.

**Table 2:** The analysis procedure purposed by Padgett (1998).

Order	Analysis Process	Analysis Method
1	Reading the recorded contents to find the meaningful units.	Open coding
2	Separating necessary and unnecessary data.	constant comparative analysis
3	Finding relationships between codes or categories to form a central theme.	Checking the association between codes
4	Linking research results with existing knowledge Are the research results consistent with the existing research results in the literature? Can it broaden the scope of our knowledge? Or does it expose existing ideas on the subject to be false?	

Researchers arranged to table the way of analyzing suggested by Padgett (1998).

**Table 3:** Analysis result.

Order	Newly Identified Factor	Factors Identified in Previous Studies
internal	curiosity	curiosity
	elevated mood and stress relief	emptiness emotional lability thrill-seeking behaviors impulse control problem
external	family (parent, grandparent, cousin)	family history family relation (family conflict, neglect, dysfunctional communication, inconsistent parenting attitude)
	friends	peer pressure rites of passage
	older friends at school	
	neighbors (adult)	recommendation of adults outside the family
	Korean traditional of alcohol making	
	workplace that recommended alcohol	
	generous drinking culture	

## Data Availability

Not applicable.
